# Artificial Intelligence and Uterine Fibroids: A Useful Combination for Diagnosis and Treatment

**DOI:** 10.3390/jcm14103454

**Published:** 2025-05-15

**Authors:** Andrea Tinelli, Andrea Morciano, Radmila Sparic, Safak Hatirnaz, Lorenzo E. Malgieri, Antonio Malvasi, Antonio D’Amato, Giorgio Maria Baldini, Giovanni Pecorella

**Affiliations:** 1 Department of Obstetrics and Gynecology, CERICSAL [CEntro di RIcerca Clinico SALentino], Veris delli Ponti Hospital, 73020 Scorrano, Lecce, Italy; giovannipecorella2690@gmail.com; 2 Department of Obstetrics and Gynecology, Cardinal Panico Hospital, 73039 Tricase, Lecce, Italy; drmorciano@gmail.com; 3 Clinic for Gynecology and Obstetrics, University Clinical Center of Serbia, 11000 Belgrade, Serbia; radmila@rcub.bg.ac.rs; 4 Faculty of Medicine, University of Belgrade, 11000 Belgrade, Serbia; 5 Mediliv Medical Center, 55100 Samsun, Türkiye; safakhatirnaz@gmail.com; 6 New European Surgical Ademy (NESA), 10117 Berlin, Germany; lorenzo@malgieri.org; 7 Unit of Obstetrics and Gynecology, Department of Interdisciplinary Medicine, Policlinico of Bari, University of Bari “Aldo Moro”, Piazza Giulio Cesare 11, 70124 Bari, Italy; antoniomalvasi@gmail.com (A.M.); antoniodamato19@libero.it (A.D.); gbaldini97@gmail.com (G.M.B.)

**Keywords:** artificial intelligence, uterine fibroids, myoma, uterine leiomyosarcoma, machine learning, deep neural network, magnetic resonance imaging, high-intensity focused ultrasound

## Abstract

This manuscript examines the role of artificial intelligence (AI) in the diagnosis and treatment of uterine fibroids and uterine sarcomas, offering a comprehensive assessment of AI-supported diagnostic and therapeutic techniques. Through the use of radiomics, machine learning, and deep neural network models, AI shows promise in identifying benign and malignant uterine lesions, directing therapeutic decisions, and improving diagnostic accuracy. It also demonstrates significant capabilities in the timely detection of fibroids. Additionally, AI improves surgical precision, real-time structure detection, and patient outcomes by transforming surgical techniques such as myomectomy, robot-assisted laparoscopic surgery, and High-Intensity Focused Ultrasound (HIFU) ablation. By helping to forecast treatment outcomes and monitor progress during procedures like uterine fibroid embolization, AI also offers a fresh and fascinating perspective for improving the clinical management of these conditions. This review critically assesses the current literature, identifies the advantages and limitations of various AI approaches, and provides future directions for research and clinical implementation.

## 1. Introduction

Uterine fibroids, also known as leiomyomas, are the most common benign tumor entities in women. The incidence ranges from 5.4% to 77% based on the analyzed population [1]. Uterine fibroid tumors impose significant costs on the healthcare system, both in terms of direct and indirect costs. Whereas direct costs are related to the price of prescription drugs, medical services, and other treatments, indirect costs are related to lost productivity. The yearly costs associated with the diagnosis or surgical excision of uterine fibroids may range from USD 11,717 to USD 25,023 per patient [2].

The smooth muscle layer (myometrium) of the uterus contains non-cancerous monoclonal tumors called fibroids that react to steroid hormones like progesterone and estrogen [3]. Significant symptoms, including pelvic discomfort, painful menstruation, excessive menstrual bleeding, anemia, urinary incontinence, recurrent miscarriages, premature labor, and infertility, are believed to affect 15 to 30 percent of women with fibroids [4].

Several non-modifiable risk factors have been identified for the development of fibroids, including age, age at menarche, and ethnicity [5]. The likelihood of fibroid development increases with age and a positive family history [6,7].

Furthermore, a history of pregnancy and lactation appears to have a protective impact on the development of fibroid tumors, whereas earlier menarche is linked to a higher risk of fibroid growth [8,9,10]. Moreover, it has been shown that black women have a higher prevalence of multiple fibroids and larger fibroid volumes [11]. Furthermore, females who have an accompanying deficiency in vitamin D typically exhibit larger fibroids [12].

When considering women of both Black and White ethnicities, the average growth rate for fibroids is 9% per six months, with variations ranging from −89% to 138%. After the age of 35, white women’s fibroids tend to grow less quickly, whereas black women do not follow this pattern [8]. Moreover, smaller fibroids, submucous fibroids, and intramural fundal fibroids exhibit a higher rate of growth [13,14]. This review addressed the use of machine learning (ML) and artificial intelligence (AI) to assist doctors in diagnosing and treating fibroids. The authors focused mainly on applications pertaining to diagnosis and treatment, as these were the domains where artificial intelligence had the potential to exert the most influence.

## 2. Methodology

### 2.1. Search Strategy and Selection Criteria

A literature review covering the period from 2019 to March 2025 was performed using a combination of keywords, such as “uterine fibroids”, “leiomyoma”, “myoma”, “uterine sarcoma”, “leiomyosarcoma”, “artificial intelligence”, “machine learning”, “deep learning”, “neural network”, “radiomics”, “computer-aided diagnosis”, “diagnosis”, “segmentation”, “classification”, “treatment”, “surgery”, “embolization”, “high-intensity focused ultrasound”, and “HIFU”. The search was limited to articles in English and studies conducted on humans published in indexed journals on PubMed/MEDLINE, Scopus, Web of Science, IEEE Xplore, and Google Scholar.

### 2.2. Inclusion and Exclusion Criteria

The inclusion criteria are as follows: (a) original research articles, systematic reviews, meta-analyses, and significant case studies; (b) studies focusing on AI applications in the diagnosis or treatment of uterine fibroids or sarcomas; (c) studies with clear methodology and results reporting; and (d) both retrospective and prospective studies.

The exclusion criteria are as follows: (i) studies focused solely on conventional imaging or treatment without an AI component; (ii) conference abstracts with insufficient methodological details; and (iii) opinion articles that do not include original data.

### 2.3. Data Extraction and Quality Assessment

For each included study, information was extracted on (a) study design and sample size; (b) AI methodology (algorithms, architectures, training approaches); (c) input data types and preprocessing methods; (d) performance metrics and validation approach; (e) clinical application; and (f) key findings, advantages, and limitations.

Quality assessment was performed using the appropriate checklist based on the study design: STROBE for observational studies, MINIMAR for AI in medical imaging studies, and PRISMA for any included systematic reviews.

### 2.4. Data Synthesis and Analysis

Due to the heterogeneity of AI methodologies, input data types, and outcome measures, a narrative synthesis approach was adopted instead of a meta-analysis. Studies were grouped based on their primary focus (diagnosis vs. treatment) and specific application areas. Comparative tables and figures were created to illustrate methodological differences, performance metrics, and clinical outcomes.

## 3. Traditional Fibroid Diagnosis

Typically, fibroids are accidentally discovered during imaging studies or routine pelvic exams performed on women who are asymptomatic. Fibroid diagnosis is primarily based on the patient’s reported symptoms, such as irregular menstrual bleeding, pelvic pain, pelvic pressure, or anemia symptoms [15]. Although transvaginal ultrasonography is quite sensitive and can identify uterine fibroids in 90% to 99% of cases, it may not detect subserosal or tiny fibroids [16,17]. The assistance provided by hysteroscopy, in addition to transvaginal sonography, further increases the detection rate of intramural fibroids [18]. Fibroids must be clearly distinguished preoperatively from the much more concerning leiomyosarcomas, and this task is not always straightforward [19].

## 4. Leiomyomas vs. Leiomyosarcomas: The Diagnostic Challenge

Gynecological sarcomas are uncommon malignant tumors that account for 3–9% of all malignant uterine tumors, with a frequency of 1.5–3 per 100,000 people. They originate from the smooth muscle cells of the myometrium, the endometrial stroma, or the uterine connective tissue. Within the category of malignant mesenchymal tumors (MMT) and malignant mixed epithelial–mesenchymal tumors, the World Health Organization (WHO) currently distinguishes between leiomyosarcoma (LMS), low-grade endometrial stromal sarcoma (LG-ESS), high-grade endometrial stromal sarcoma (HG-ESS), undifferentiated uterine sarcoma (UUS), and adenosarcoma (AS) [20].

Moreover, there exist smooth muscle tumors of unknown malignant potential (STUMP), which comprise tumors that lack a clear classification as either benign leiomyomas or malignant leiomyosarcomas [21]. Leiomyosarcomas are the most prevalent type of uterine sarcoma, constituting 60 to 70% of cases [22]. Alarmingly, approximately 0.1 to 0.3% of patients who undergo surgery with a diagnosis of uterine leiomyomas are estimated to actually have a uterine sarcoma [23]. Laboratory tests are not routinely used to differentiate uterine sarcomas from fibroids. Some studies have shown that serum lactate dehydrogenase (LDH) levels [24], along with dynamic magnetic resonance imaging (MRI), may aid in distinguishing uterine sarcomas from fibroids. The combination of LDH, D-dimer, and C-reactive protein can help differentiate sarcomas from degenerated or atypical leiomyomas [25].

The screening method for uterine fibroids vs. leiomyosarcomas was represented by ultrasound. Kohler et al. [26] observed a notable discrepancy in sonographic findings between leiomyomas (10%) and leiomyosarcomas (81%) (*p* < 0.001). A “sonographic suspicious” was identified based on several criteria: poorly defined borders with the myometrium, predominantly heterogeneous echogenicity with pronounced areas of hyperechogenicity, patchy or predominantly hypo-to-anechoic regions within the tumor, irregular borders between areas of different echogenicity, and signs of serosal involvement or rupture [26]. However, more research is needed to confirm their reliability in preoperative evaluation.

## 5. Radiomics in Uterine Fibroid Diagnosis

**Figure 1:**
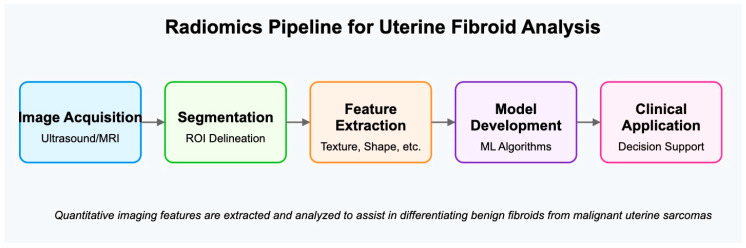
Radiomics pipeline for uterine fibroid analysis illustrated by a diagram showing the following sequence: Image Acquisition → Segmentation → Feature Extraction → Model Development → Clinical Application.

Radiomics represents a dynamic area of research focused on quantitatively analyzing medical images to extract radiomic features that capture tissue and lesion characteristics like heterogeneity and shape. When combined with demographic, histologic, genomic, or proteomic data, these features can aid in clinical decision-making and improve diagnostic accuracy (Figure 1) [27].

### 5.1. Pioneering Studies and Comparative Assessment

Three notable studies applied radiomics and AI to uterine fibroid diagnosis, each with varying approaches, strengths, and limitations.

Chiappa et al. aimed to develop and evaluate a radiomics and machine learning model applied to ultrasound (US) images for predicting the risk of malignancy in uterine mesenchymal lesions [28]. This single-center retrospective study included patients who underwent surgery for malignant uterine mesenchymal lesions (sarcoma) and a control group with benign lesions (fibroid). Their methodology involved radiomics analysis on preoperative US images according to the International Biomarker Standardization Initiative guidelines, utilizing the TRACE4 radiomic platform to create, validate, and test a classification model for differential diagnosis. The study included 70 women with uterine mesenchymal lesions (20 sarcomas, 50 fibroids), extracting 319 radiomics features, of which 308 were identified as stable. Various machine learning classifiers were developed, with the best model demonstrating an accuracy of 0.85 ± 0.01, sensitivity of 0.80 ± 0.01, specificity of 0.87 ± 0.01, and AUC of 0.86 ± 0.03. While promising, this study faced limitations due to its relatively small sample size and single-center design, which may introduce selection bias and limit generalizability to diverse populations.

In contrast, Huo et al. employed a deep learning approach, creating an AI-supported method to enhance the diagnostic accuracy of junior ultrasonographers in identifying uterine fibroids [29]. Their study involved a substantially larger dataset of 3870 ultrasound images from 667 patients with fibroids and 570 women without uterine lesions. Using a deep convolutional neural network (DCNN) model, they demonstrated that AI assistance significantly improved junior ultrasonographers’ diagnostic performance (accuracy: 94.72% vs. 86.63%, *p* < 0.001; sensitivity: 92.82% vs. 83.21%, *p* = 0.001; specificity: 97.05% vs. 90.80%, *p* = 0.009), elevating it to levels comparable with that of senior specialists.

Yang et al. took a different approach by adapting the You Only Look Once, Version 3 (YOLOv3) object detection framework to identify fibroids in ultrasound images, achieving an impressive precision of 98.38% [30,31]. This represented a novel adaptation of technology originally designed for general object detection in videos and images to the specific medical context of fibroid identification.

The comparison among the three studies is reported in Table 1.

### 5.2. Methodological Differences and Their Implications

These studies differed significantly in their fundamental approaches to AI application. Chiappa’s radiomics model required explicit feature extraction and selection, which provided potentially greater interpretability but demanded more domain expertise in feature engineering. The 308 stable features they identified represented quantifiable characteristics of the images that might correspond to clinically relevant tissue properties.

Conversely, Huo’s DCNN approach utilized deep learning to automatically learn relevant features from the images without manual feature engineering. While potentially more powerful in capturing complex patterns, this “black box” approach offered less transparency regarding which specific image characteristics drive the classification decisions. This interpretability challenge represented a significant limitation for clinical adoption, as physicians typically prefer to understand the rationale behind diagnostic suggestions.

The sample size disparity between these studies (70 patients in Chiappa vs. 1237 patients in Huo) raises important considerations about statistical power and generalizability. Larger datasets typically produce more robust models capable of handling greater patient diversity, giving Huo’s study an advantage in this aspect. However, Chiappa’s focus on the challenging differentiation between fibroids and sarcomas addresses a more clinically significant diagnostic dilemma than Huo’s focus on simply identifying the presence of fibroids.

**Figure 2:**
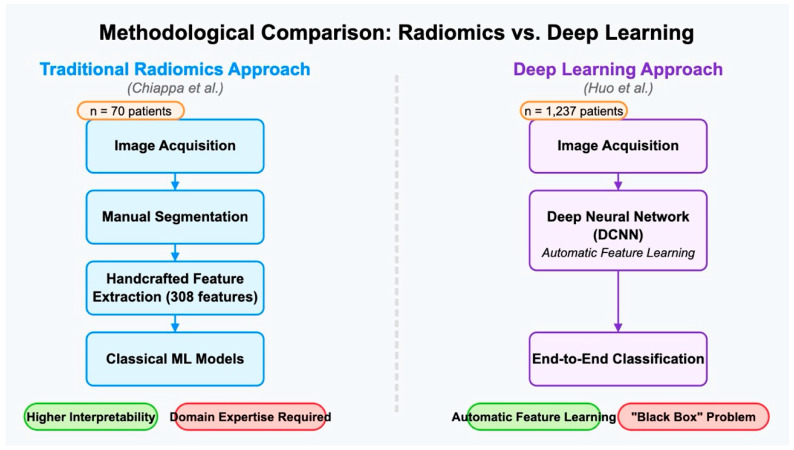
Methodological comparison between traditional radiomics and deep learning approaches illustrated by a diagram comparing the feature engineering in radiomics with the automatic feature learning in deep learning [28,29].

The methodological differentiation among the three studies is reported in Figure 2.

### 5.3. Clinical Integration and Practical Value

The clinical utility of these approaches varied considerably. Huo’s study demonstrated a tangible benefit by showing how AI assistance could elevate junior practitioners’ diagnostic abilities to match those of experienced specialists. This had immediate practical implications for healthcare delivery, particularly in settings with limited access to specialist expertise. The authors validated this improvement through direct comparison with senior ultrasonographers, providing a clear benchmark for the technology’s effectiveness.

Chiappa’s work, while addressing the critical clinical challenge of distinguishing benign from malignant lesions, did not demonstrate how their model would integrate into clinical workflows or how it would compare with specialists’ performance. This limited assessment of its practical value, despite addressing a clinically important question.

### 5.4. Limitations and Future Directions

Several limitations affected the current state of AI applications in uterine fibroid diagnosis. The most significant limitations included data diversity challenges, interpretability issues, validation gaps, concerns regarding rare variant performance, and implementation barriers.

Most studies, including Chiappa’s, relied on single-center data, limiting generalizability across different patient populations, imaging equipment, and clinical settings. Future research should incorporate multi-center, multi-ethnic data to develop more robust models.

Deep learning approaches like Huo’s DCNN suffer from limited interpretability, making it difficult for clinicians to understand and trust the reasoning behind AI predictions. Hybrid approaches that combine the interpretability of radiomics with the power of deep learning merit exploration. External validation on independent datasets has remained insufficient throughout most studies, raising questions about real-world performance. Prospective studies are needed to confirm retrospective findings.

Current models focus primarily on common presentations, with limited evidence of effectiveness for rare variants of fibroids or unusual imaging presentations of sarcomas. Specialized models or approaches for these edge cases require development. Technical requirements, integration with existing Picture Archiving and Communication System (PACS) systems, training needs, and cost-effectiveness concerns present practical challenges to clinical adoption that remain largely unaddressed in current research.

Future research should focus on addressing these limitations while working toward standardized protocols for model development, validation, and implementation. Multi-institutional collaborations that collect diverse datasets could significantly advance the field, as could efforts to develop more interpretable AI systems that align with clinical decision-making processes.

## 6. MRI-Guided Traditional Fibroid Diagnosis

When there is uncertainty and sonography is unable to rule out malignant leiomyosarcomas, despite the fact that they exhibit similar clinical presentations, MRI is crucial in making this distinction [32]. This paragraph details MRI-guided diagnosis, which is used to accurately distinguish between these entities, highlighting the importance of particular imaging characteristics and their implications for clinical practice. When contrast medium is added, uterine sarcomas can be diagnosed more accurately; sarcomas originating from the myometrium usually show heterogeneous enhancement. Prior research has noted that central unenhanced regions on MRI are strongly suggestive of sarcomas, both in terms of sensitivity and specificity. Distinctive enhancement attributes and dynamic enhancement curves further facilitate the differentiation of sarcomas from leiomyomas [33,34].

Subacute hemorrhage produces high T1 signal intensity, distinguishing sarcomas from leiomyomas. The identification of blood products within the lesion, particularly in the absence of typical hormonal insults, is highly suggestive of sarcoma. Susceptibility-weighted imaging (SWI) can detect areas of previous hemorrhage, providing additional diagnostic value [35,36]. Low T2 signal intensity dark areas are indicative of flow voids or intralesional hemosiderin, contributing to the diagnosis of leiomyosarcomas. High T2 signal intensity is present in both sarcomas and degenerating leiomyomas, necessitating correlation with T1WI and enhancement for accurate interpretation [37].

Endometrial involvement is a characteristic feature of adenosarcomas, endometrial stromal sarcomas (ESS), and leiomyosarcomas. Assessing the lesion’s outline with the endometrium is crucial, as sarcomas can involve the endometrium in a significant proportion of cases [38].

High diffusion-weighted imaging (DWI) signal intensity and low apparent diffusion coefficient (ADC) values are indicative of leiomyosarcomas, although overlap is observed in cellular leiomyomas. Correlating restricted diffusion areas with T2 and T1WI findings is imperative to avoid misinterpretation [39,40,41]. Nakagawa et al. [42] distinguished between leiomyomas and uterine sarcomas using the T2-weighted MRI screening method and a machine learning approach. Eighty individuals who underwent pelvic 3T MRI examinations were included in the retrospective investigation (30 with uterine sarcoma and 50 with benign leiomyoma). To create prediction models based on 12 textural parameters from T1WI and T2WI, apparent diffusion coefficient maps, contrast-enhanced T1WI, tumor size, and age, six machine learning algorithms were used. Areas under the curve (AUCs) were calculated using receiver operating characteristic analysis for each model via 10-fold cross-validation and were compared to those of two board-certified radiologists. Age was found to be the most significant factor for differentiating between the two groups. The AUC for eXtreme Gradient Boosting was significantly higher than those for both radiologists (0.93 vs. 0.80 and 0.68, *p* = 0.03 and *p* < 0.001, respectively). According to the study, machine learning techniques outperformed experienced radiologists in distinguishing uterine sarcomas from leiomyomas with high signal intensity on T2WI [42].

## 7. Artificial Intelligence Support in Fibroid Diagnosis

The use of AI in medical diagnostics has emerged as a potentially transformative approach to the challenging process of differentiating between uterine leiomyomas and sarcomas. Several methodologically distinct studies have demonstrated promising results, although they also have important limitations and varying levels of clinical applicability.

### 7.1. Deep Neural Network Approaches

Toyohara et al. [19] conducted a pioneering study employing deep neural network (DNN) models to improve diagnostic accuracy for uterine sarcoma cases using MRI. The research utilized fifteen distinct MRI sequences, with tumor margins and degeneration carefully assessed by six radiologists according to strict standards. The methodology was comprehensive, capturing imaging conditions for each MRI sequence to ensure reliability and consistency. After the DICOM data was transformed into normalized JPEG format, the datasets were split into six groups for cross-validation, ensuring model robustness during training [19].

The findings were impressive, with DNN models achieving 90.3% accuracy, 89.8% sensitivity, and 91.7% specificity—performance comparable to that of radiological specialists. Furthermore, the DNN models demonstrated the ability to improve diagnostic precision by outperforming professionals in terms of SS-Avg and sensitivity [33,43]. The MobileNet-V2 network architecture was employed, consisting of 88 layers with 3,538,984 learning parameters, providing a solid foundation for the AI-driven analysis of uterine cancers.

The study’s strengths included its comprehensive methodology, which utilized multiple MRI sequences and a rigorous validation approach. However, significant limitations must be acknowledged: despite being one of the largest studies in this domain, the sample included only 63 sarcoma cases, which may be insufficient for developing a robust model given the heterogeneity of uterine sarcomas. Additionally, while the model performed well in controlled evaluations, its real-world clinical integration was not tested.

In a different but complementary approach, Huo et al. [29] developed a DCNN to enhance the diagnostic accuracy of junior ultrasonographers in identifying uterine fibroids. This study leveraged a substantially larger dataset of 3870 ultrasound images from 1237 patients, representing a significant advantage in terms of statistical power. The DCNN model significantly improved junior ultrasonographers’ performance (accuracy: 94.72% vs. 86.63%, *p* < 0.001), effectively elevating their diagnostic abilities to match those of senior specialists.

A key distinction of this study was its direct evaluation of clinical integration, demonstrating tangible improvements in diagnostic performance rather than merely comparing AI to human performance in isolation. However, the study focused primarily on fibroid detection rather than on the more challenging task of distinguishing between fibroids and sarcomas, which limits its direct applicability to the critical clinical challenge of preoperative sarcoma diagnosis.

### 7.2. Radiomics and Machine Learning Approaches

In contrast to deep learning approaches, Chiappa et al. [28] employed radiomics with machine learning rather than deep learning. Their ADMIRAL pilot study included a relatively small sample of 70 women with uterine mesenchymal lesions (20 sarcomas and 50 fibroids). Using the International Biomarker Standardization Initiative guidelines, they extracted 319 radiomics features from ultrasound images, with 308 features identified as stable. Various machine learning classifiers were developed, with the best model achieving an accuracy of 0.85 ± 0.01, a sensitivity of 0.80 ± 0.01, a specificity of 0.87 ± 0.01, and an AUC of 0.86 ± 0.03.

The radiomics approach offered potentially greater interpretability through explicit feature extraction and selection but required more domain expertise in feature engineering. The study’s primary limitations included its single-center design and small sample size, which could introduce selection bias and limit generalizability to diverse populations.

Malek et al. [44] took yet another approach, utilizing perfusion-weighted magnetic resonance imaging (PWI) with a decision tree ensemble classifier. Forty-two women with a total of 60 masses (10 uterine sarcomas and 50 benign leiomyomas) were included, with two manually defined regions of interest analyzed for each mass. An analysis of the postoperative histopathology corroborated the reference diagnosis. A 3-Tesla MR imager was used to perform MRI protocols, which included PWI. For each mass, two manually defined regions of interest (ROIs) were created: one that included the whole tumor and another that concentrated on the region showing the greatest contrast enhancement. For comparison, additional ROIs were placed on the myometrium and psoas muscle. The ROIs were analyzed using the DCE Tool plug-in inside the ClearCanvas framework, and seven parameters were extracted for contrast uptake modeling using the modified Tofts model. After that, a decision tree ensemble was trained with the retrieved parameters to categorize lesions as benign or malignant. The classifier’s performance was assessed using leave-one-out cross-validation (LOOCV). The metrics obtained from ROI L or ROI S between benign leiomyomas and uterine sarcoma did not differ significantly, according to the results (all *p* > 0.05). By providing the classifier with seven characteristics from ROI L, an overall accuracy of 66.7% was attained. When 21 features from ROI L, ROI M, and ROI P were included, the accuracy of the classifier increased, although the exact value was not provided. While no individual parameter significantly differentiated between benign and malignant lesions, when combined through the machine learning classifier, promising discriminative power was achieved, with reported sensitivity of 100% and specificity of 90% at the optimal operating point. This study highlighted the value of multiparametric analysis but was limited by an extremely small sample of sarcoma cases (n = 10), raising serious concerns about statistical power and generalizability [44].

### 7.3. Comparative Analysis and Methodological Considerations

The approaches described above differed fundamentally in several ways that merit critical comparison. There was substantial variation in sample size between studies, ranging from Malek’s 42 patients to Huo’s 1237 patients. This variation affected model reliability, with larger samples typically producing more robust models capable of handling greater patient diversity. The small sarcoma cohorts in all studies (with a maximum of 63 cases in Toyohara) remained problematic given the heterogeneity of uterine sarcomas.

While radiomics approaches (by Chiappa and Malek) required explicit feature extraction based on domain knowledge, deep learning approaches (by Toyohara and Huo) automatically learned relevant features from images. This represented a fundamental trade-off between interpretability and the potential for higher pattern recognition capability.

Studies employed different imaging techniques—ultrasound (by Chiappa and Huo) and MRI (by Toyohara and Malek)—which have associated differences in accessibility, cost, and the types of visual information captured. The optimal modality for AI-assisted diagnosis remains unclear.

Cross-validation approaches varied, with most studies employing only internal validation. The absence of external validation on independent datasets from different institutions represented a significant limitation across all studies, raising questions about their generalizability to real-world clinical settings.

Huo’s study stood out for directly demonstrating how AI assistance improved clinical performance, while other studies primarily compared AI to human diagnosticians rather than showing how they might work together effectively.

**Figure 3:**
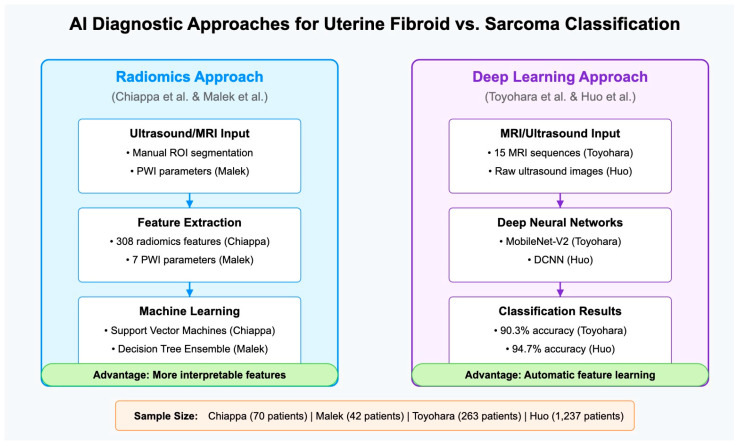
Comparison of radiomics-based approaches (**left**) [28,44], and deep learning approaches (**right**) [19,29] in the diagnosis of uterine fibroids versus sarcomas. Radiomics approaches require manual feature extraction but offer greater interpretability, while deep learning methods automatically learn features directly from images but function as “black boxes”.

The comparative analysis is shown in Figure 3.

### 7.4. Clinical Implications and Future Directions

Despite promising results, several significant limitations must be addressed before widespread clinical adoption can be recommended.

Most studies relied on single-center data, limiting generalizability across different patient populations, imaging equipment, and clinical settings. Multi-institutional studies with diverse patient cohorts are needed.

Deep learning approaches suffer from limited interpretability, making it difficult for clinicians to understand and trust the reasoning behind AI predictions. This “black box” problem remains a significant hurdle for clinical adoption.

Current models predominantly focus on common presentations, with limited evidence regarding their effectiveness for rare variants or unusual imaging presentations. Given that uterine sarcomas are already rare entities, their unusual variants present an even greater challenge.

Technical requirements, integration with existing PACS systems, training needs, and cost-effectiveness concerns presented practical challenges to clinical adoption that remained largely unaddressed.

Most importantly, prospective studies in clinical settings are required to confirm the promising findings of these retrospective analyses.

In conclusion, while AI approaches to uterine fibroid and sarcoma diagnosis show considerable promise, significant methodological differences between studies and persistent limitations in data diversity, interpretability, and clinical integration highlight the need for continued research. The most promising path forward appears to lie in combining the strengths of different approaches—perhaps integrating the interpretability of radiomics with the pattern-recognition power of deep learning—while addressing implementation challenges through close collaboration among AI developers, radiologists, and gynecologists.

## 8. Alternative AI Powered Diagnostic Methods

While image-based AI systems for fibroid detection showed promising results, alternative approaches leveraging other sensing modalities and feedback mechanisms represent an important complementary direction for research. These methods could potentially overcome the limitations of conventional imaging techniques, particularly in robotically assisted surgical scenarios where direct tactile feedback is lost.

### 8.1. Haptic Interface Systems for Fibroid Localization

Doria et al. [45] developed a pioneering approach that integrated tactile feedback to improve lump localization in Robot-Assisted Minimally Invasive Surgery (RMIS), specifically targeting uterine fibroids. Their work addressed a critical gap in current robotic surgical systems, which typically lack the tactile sensation that surgeons naturally employ during traditional open procedures to differentiate between normal tissue and fibroids.

**Figure 4:**
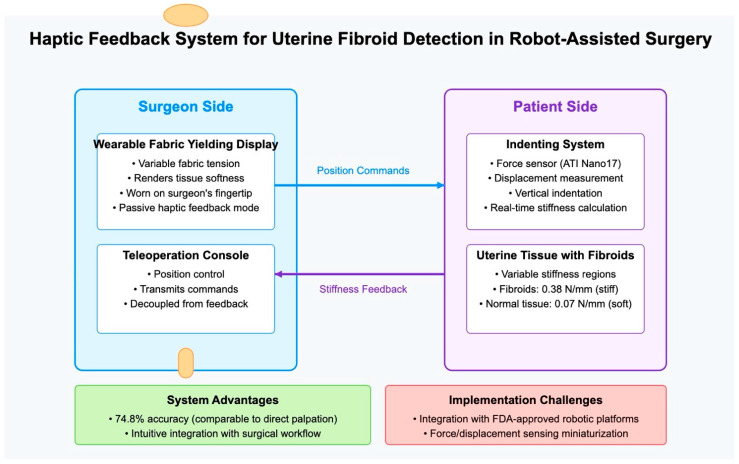
Haptic feedback system for uterine fibroid detection, developed by Doria et al. [45].

Figure 4 illustrates the haptic feedback system developed by Doria et al. [45], showing: (a) Surgeon Side: the teleoperating console with the Wearable Fabric Yielding Display (W-FYD), which renders tissue softness through variable fabric tension on the surgeon’s fingertip; (b) Patient Side: the indenting system that measures tissue stiffness through force sensors and displacement measurement, interacting with uterine tissue containing fibroids; (c) Data Flow: position commands flowing from the surgeon side to the patient side, with stiffness feedback returning to provide tactile information; (d) Key Parameters: stiffness values for fibroids (0.38 N/mm) compared to normal tissue (0.07 N/mm); and (e) System Evaluation: key advantages and implementation challenges.

This visual representation helps clarify the innovative approach to haptic feedback in robot-assisted surgery for fibroid detection, emphasizing how it complements existing image-based diagnostic methods.

The system employed a Wearable Fabric Yielding Display (W-FYD) in a teleoperation architecture that simulated robot-assisted surgical palpation of leiomyomas. The W-FYD consisted of a fabric interface whose stretching state could be modulated by controlling two DC motors, allowing the device to render different softness characteristics to the surgeon’s fingertip. This approach built on the established knowledge that fibroids typically appear as hard lumps within uterine tissues, exhibiting significantly higher stiffness compared to the surrounding tissue.

The researchers performed a comprehensive validation of their approach: (a) first conducting ex vivo characterization of real uterine tissues with embedded fibroids to establish the stiffness workspace; (b) developing silicone specimens that accurately replicated the mechanical properties of uterine tissues and fibroids; and (c) testing with 13 gynecologic surgeons in simulated palpation tasks.

Their experimental results demonstrated that the haptic feedback system enabled fibroid discrimination with accuracy comparable to direct manual palpation. Specifically, surgeons achieved 74.8% accuracy using the teleoperation system with integrated tactile feedback, compared to 83.8% with direct finger palpation, with no statistically significant difference between the methods. The surgeons reported that the haptic feedback was intuitive, comfortable, and highly effective for fibroid localization.

### 8.2. Comparison of Haptic-Based Approach with Image-Based AI Approach

The haptic-based approach offers several distinct advantages compared to the image-based AI systems (Table 2) discussed in previous sections.

While radiomics and deep learning methods (Chiappa et al. [28], Huo et al. [29], Toyohara et al. [19], Malek et al. [44]) extract features from visual data, the haptic approach captures mechanical tissue properties that may not be visible in images.

Unlike preoperative diagnostic systems, the haptic interface was designed for real-time use during surgery, enabling continuous assessment as tissue was manipulated. Rather than providing a separate diagnostic output, the system seamlessly integrated into the surgical workflow by augmenting the surgeon’s existing sensory capabilities.

The performance of the system was not affected by image acquisition parameters or artifacts that can limit image-based approaches. However, the haptic approach also faced significant implementation challenges: the integration complexity with FDA-approved robotic platforms required extensive development and validation; translating the approach to clinical practice necessitated the development of miniaturized force and displacement sensing for the robotic instruments; and reliable haptic rendering required accurate stiffness measurement and appropriate scaling to match human perception capabilities.

### 8.3. Future Directions

The integration of haptic feedback with AI-based analysis represents a promising frontier. Future systems might combine real-time tissue stiffness data acquired during robotic surgery with machine learning algorithms that continuously learn from surgical experiences to improve fibroid detection accuracy.

A hybrid approach that incorporates both visual and haptic information processed through multimodal AI could potentially outperform either modality alone, particularly in challenging cases in which fibroids are difficult to distinguish visually or are embedded deep within the myometrium. The development of AI systems capable of processing and interpreting haptic data in conjunction with visual information could represent the next evolutionary step in computer-assisted fibroid diagnosis and surgical navigation.

Other emerging approaches include piezoelectric tactile sensors for direct tissue stiffness sensing, soft electronic sensors for clinical palpation, and systems that integrate optical sensing with haptic feedback to enhance tissue characterization during minimally invasive procedures.

These alternative modalities complement traditional imaging-based diagnostic methods, potentially enhancing both preoperative planning and intraoperative decision-making in the management of uterine fibroids.

## 9. Uterine Fibroid Treatment

Among the medications used in medical therapy to treat severe menstrual bleeding are nonsteroidal anti-inflammatory drugs, tranexamic acid, and hormonal contraceptives. Gonadotropin-releasing hormone agonists and antagonists, as well as selective progesterone receptor modulators, are good choices for patients who need relief from symptoms prior to surgery or who are approaching menopause. Surgical procedures such as myomectomy, hysterectomy, uterine artery embolization, and MRI-guided targeted ultrasound surgery provide additional therapeutic options. Each technique has its own indications and considerations, which emphasize the significance of a customized treatment plan informed by clinical knowledge and patient needs [46]. The authors focused on the role of AI in fibroid therapy.

## 10. Traditional Surgery

Every surgeon is aware of the crucial role played by the pseudocapsule in myomectomy. However, they also face the challenge of locating the precise layer in which it lies [47]. It seems that artificial intelligence can help with this problem. The objective of Török et al. [48] was to assess how well a deep neural network distinguished between fibroids and normal myometrium following surgery. The network was tested with 1600 unobserved photos following training on 4688 images. Fibroid resection was the surgical procedure performed. Thirteen instances of transcervical myomectomy videos were used. To recognize labeled structures, the fully convolutional neural network (FCNN) used a variety of filters and techniques. Using the Hausdorff measure, the FCNN that was trained on manually annotated images was able to separate normal myometrium with 86.19% pixel-wise accuracy. In summary, utilizing deep learning techniques for analyzing endoscopic video frames showed promise for the real-time identification of structures during endoscopic surgery [48].

The Török approach represents an important step toward AI-enhanced traditional surgery that preserves the fundamental surgical approach while addressing one of its key limitations—the identification of optimal tissue planes in the absence of tactile feedback. This FCNN approach to myoma–myometrium segmentation processed an input hysteroscopic image, compared it with manual annotations for training, and produced automated segmentation results showing myoma (white) and normal myometrium (black), which could then be overlaid on the original image for surgical guidance.

Several technical refinements could enhance clinical utility: integration with stereoscopic visualization systems to provide depth perception and tissue differentiation; real-time marking of surgical margins to guide the extent of resection; optimization for lower-powered computing platforms to enable practical implementation in typical operating room environments; and expansion of the training dataset to include diverse pathologies and surgical scenarios.

As summarized by the authors, “the experiences of an expert endoscopist could be simulated and reproduced through the application of FCNNs”. This represents a key concept in AI-enhanced surgery: capturing expert-level pattern recognition and making it broadly available to surgeons of all experience levels.

## 11. Robot-Assisted Surgery

In the realm of surgery, artificial intelligence is demonstrating its value in intricate cases, such as the one detailed by Mercorio in a recent case study. The intraoperative application of 3D imaging reconstruction for a difficult multiple myomectomy procedure carried out via robot-assisted laparoscopic surgery is described in this research. With more than 20 fibroids, the patient presented an unusual case of a symptomatic fibromatous uterus. Real-time 3D reconstruction technology was used during the procedure to help modify the surgical approach. With only 105 min of surgery, 21 fibroids were removed, and there was minimal intraoperative blood loss of 150 mL, resulting in an astounding outcome. The patient was discharged the next day. By utilizing 3D imaging technology, this innovative use of AI addresses a drawback of robot-assisted surgery, namely the absence of tactile input. With the aid of intraoperative guidance, this method enables surgeons to quickly identify fibroids and optimize the rebuilding process [49].

## 12. High-Intensity Focused Ultrasound (HIFU)

Among the various treatment modalities for uterine fibroids, High-Intensity Focused Ultrasound (HIFU) has emerged as a non-invasive approach in which artificial intelligence applications have shown significant impact. This section explores the integration of AI with HIFU for uterine fibroid treatment, examining various machine learning approaches that enhance treatment planning, execution, and outcome prediction.

### 12.1. Predictive Modeling for HIFU Treatment Outcomes

Predicting the efficacy of HIFU ablation is crucial for appropriate patient selection and treatment planning. Akpinar et al. [50] conducted a comprehensive study investigating ML models to predict treatment outcomes, specifically targeting an immediate Non-Perfused Volume (NPV) ratio of at least 90%, which serves as a key indicator of successful ablation.

Their study employed multiple ML classifiers to analyze multiparametric MRI (mp-MRI) features. Seventy-three women who underwent HIFU were divided into two groups based on the NPV ratio (≥90% and <90%). The researchers evaluated the relationship between mp-MRI features and treatment outcomes using various ML algorithms, as shown in Table 3.

The Gradient Boosting Machine (GBM) classifier demonstrated superior predictive performance (AUROC: 0.95, accuracy: 0.92) by incorporating the most informative features from each mp-MRI group. The key features identified included the Ktrans ratio of fibroid to myometrium; the ratio of the area under the curve of fibroid to myometrium; subcutaneous fat thickness; the ratio of the apparent diffusion coefficient value of fibroid to myometrium; and the T2-signal intensity of the fibroid.

These findings highlight the potential of ML algorithms to assist clinicians in patient selection for HIFU treatment, enabling more personalized and effective interventions while minimizing unsuccessful procedures.

### 12.2. Gadolinium-Free Monitoring Using Deep Learning

A significant challenge in HIFU treatment was the inability to repeatedly monitor treatment progression without administering multiple doses of gadolinium contrast agents. Slotman et al. [51] addressed this limitation by developing an innovative deep learning-based method that converted DWI into synthetic contrast-enhanced t1-weighted imaging (CE-T1WI) scans.

Their retrospective study analyzed data from 53 patients who underwent MR-HIFU treatment for uterine fibroids. The synthetic CE-T1w images were generated using a deep learning network trained on paired DWI and reference CE-T1w scans acquired during the treatment.

The quantitative analysis revealed the Dice coefficient for NPVs was 71% (±22%); the mean difference in NPV ratio between synthetic and reference scans was 1.4% (±22%), with no statistical significance (*p* = 0.79); and the absolute agreement among radiologists evaluating technical treatment success on synthetic versus reference CE-T1w scans was 83%.

This approach enabled gadolinium-free visualization of predicted NPVs, offering a solution for monitoring treatment progression during MR-HIFU therapy without the need for repeated contrast agent administration. The technique maintained similar diagnostic accuracy while eliminating the risks associated with multiple gadolinium administrations.

### 12.3. Machine Learning for Patient Selection and Efficacy Prediction

Yang et al. [52] developed and validated machine learning models to accurately predict HIFU ablation efficacy for uterine fibroids, facilitating preoperative patient selection. Their study collected data from 1000 patients who underwent ultrasound-guided HIFU ablation for uterine fibroids.

Using Least Absolute Shrinkage and Selection Operator (LASSO) regression for feature screening and five different machine learning algorithms (logistic regression, random forest, extreme gradient boosting (XGBoost), artificial neural network, and gradient boosting decision tree), they constructed predictive models for HIFU ablation efficacy.

The XGBoost model demonstrated superior performance, achieving an AUC of 0.692 in the testing dataset. Four key predictors were identified: T2-weighted image signal intensity; distance from the ventral side of uterine fibroids to the skin; platelet count; and contrast-enhanced T1-weighted imaging.

This model offers a practical tool for preoperative evaluation, potentially improving patient selection and treatment outcomes.

### 12.4. Multimodal MRI Radiomics with Deep Learning-Based Segmentation

Wen et al. [53] evaluated the effectiveness of a multimodal MRI radiomics stacking ensemble learning model that integrates CE-T1WI and Combined T2-Weighted Imaging (T2WI) with deep learning-based automatic segmentation for the preoperative prediction of HIFU ablation prognosis.

Their retrospective study collected data from 360 patients across multiple centers who underwent HIFU treatment. Automated segmentation of uterine fibroids was performed using V-net deep learning models. Radiomics features were extracted from T2WI and CE-T1WI, followed by feature selection using *t*-tests, Pearson correlation, and LASSO regression. The stacking ensemble model, which integrated Support Vector Machine (SVM), random forest (RF), Light Gradient Boosting Machine (LightGBM), and Multi-Layer Perceptron (MLP) algorithms, achieved remarkable performance: an AUC of 0.897 in the internal test set and an AUC of 0.854 in the external test set.

This approach demonstrates significantly higher predictive accuracy compared to individual base models and clinical assessments, providing a robust tool for pretreatment evaluation.

### 12.5. Deep Learning for Segmentation and 3D Reconstruction

Wang et al. [54] introduced deep learning-based nnU-Net models for the cost-effective segmentation of uterine fibroids using preoperative MRI images, followed by 3D reconstruction to guide HIFU surgery. Their approach focused on enhancing the safety and effectiveness of HIFU procedures through improved visualization and planning.

The study utilized a dataset of 550 MR images and demonstrated that the 3D nnU-Net model achieved excellent performance in segmenting uterine fibroids and surrounding structures: 92.55% Dice Similarity Coefficient (DSC) for the uterus; 95.63% DSC for uterine fibroids; 89.63% DSC for the endometrium; 97.75% DSC for the bladder; and 90.45% DSC for the urethral orifice.

This segmentation approach, when coupled with 3D reconstruction, provides surgeons with comprehensive spatial information that enhanced treatment planning and execution. The integration of this technology with intraoperative ultrasound imaging holds substantial potential for improving the safety and efficacy of HIFU procedures.

Slotman et al. [55] devised and assessed a deep learning-driven segmentation algorithm aimed at the volumetric measurement of the uterus, uterine fibroids, and non-pathological volumes (NPVs) in magnetic resonance imaging (MRI), with the objective of automatically quantifying the NPV-to-tissue fluid load (TFL), utilizing expert manual segmentations from MRI scans of 115 patients with uterine fibroids, who were either screened for or undergoing magnetic resonance high-intensity focused ultrasound (MR-HIFU) treatment. The computational framework encompassed three distinct neural networks, each designated for a particular target structure. The initial phase of the framework involved the segmentation of the uterus from contrast-enhanced T1-weighted (CE-T1w) scans. This segmentation was thereafter employed to exclude non-uterine background tissue for the subsequent segmentation of NPVs and fibroids. In the ensuing stage, NPVs were delineated from uterus-exclusive CE-T1w scans. Ultimately, fibroids were segmented from uterus-only T2-weighted (T2w) scans. The resulting segmentations facilitated the volumetric calculations for each structure. The reliability and concordance between manual and automated segmentations, as well as the corresponding volumes and NPV/TFL ratios, were meticulously evaluated. The algorithm articulated in this investigation autonomously computed the volume of the uterus, the load of fibroids, and the NPVs, potentially enabling a more objective quantification of treatment outcomes following MR-HIFU interventions for uterine fibroids, in contrast to conventional visual assessments.

### 12.6. Super-Resolution DWI Radiomics Model

Li et al. [56] assessed the feasibility and efficacy of a deep learning-based three-dimensional Super-Resolution Diffusion-Weighted Imaging (SR-DWI) radiomics model in predicting HIFU ablation prognosis. Their multicenter study included 360 patients who received HIFU treatment for uterine fibroids.

The SR-DWI approach enhanced the resolution of conventional DWI, allowing for more detailed visualization of fibroid tissue characteristics. When combined with radiomics analysis, this technique demonstrated superior predictive performance: an AUC of 0.876 in internal validation and an AUC of 0.800 in external validation.

Compared to conventional high-resolution DWI and expert radiologist assessment, the SR-DWI radiomics model showed significantly better discriminative ability. This technique effectively overcame the inherent limitations of conventional DWI, particularly addressing the anisotropic spatial resolution that affects feature extraction and analysis.

### 12.7. Comparative Analysis and Clinical Implications

**Figure 5:**
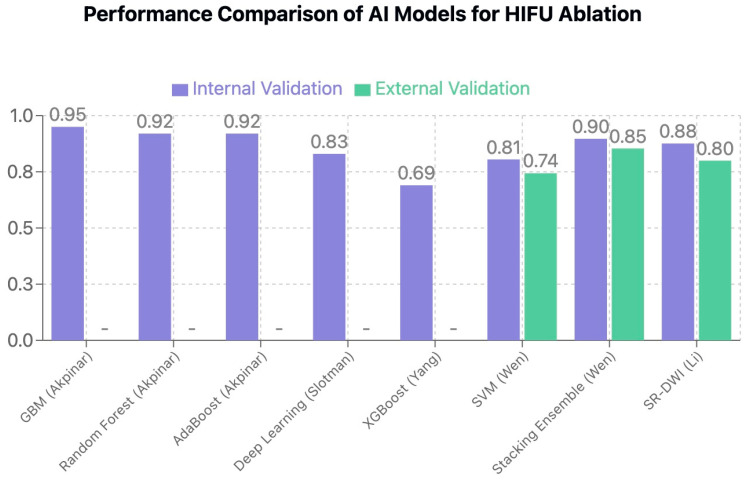
Comparison of AUC values across different AI models for HIFU ablation prediction. Higher values indicate better predictive performance.

When comparing the various AI approaches for HIFU treatment of uterine fibroids, several patterns emerged (Figure 5). The integration of AI with HIFU offers multiple advantages over traditional assessment methods.

ML models identified patients most likely to benefit from HIFU treatment with greater accuracy than conventional clinical assessment alone. Deep learning-based synthetic imaging techniques enabled gadolinium-free monitoring of treatment progression, facilitating immediate adjustments to improve outcomes. Automated segmentation and 3D reconstruction provided more precise delineation of target tissues and surrounding structures, enhancing treatment accuracy and safety. Advanced radiomics models offered superior prognostic information compared to conventional imaging assessments, enabling better patient counseling and management. By identifying key features that influenced treatment efficacy, AI models guided parameter selection and treatment protocol optimization.

These applications collectively addressed critical challenges in HIFU treatment, including patient selection, real-time monitoring, and outcome prediction, potentially expanding the applicability and effectiveness of this non-invasive treatment modality.

All these patterns of AI models are described and compared in Figure 5.

### 12.8. Study Limitations and Future Directions of Research on AI Platforms

Despite the promising results, several limitations were acknowledged: most studies had relatively small sample sizes from limited centers, which might affect model generalizability; many deep learning approaches functioned as “black boxes,” making clinical interpretation and integration challenging; prospective multicenter validation studies are needed to confirm the clinical utility of these AI applications; and variations in imaging protocols, segmentation techniques, and feature extraction methods limited direct comparisons between studies.

Future research should focus on developing integrated AI platforms that combine patient selection, treatment planning, real-time monitoring, and outcome prediction within a unified framework; exploring multimodal approaches that leverage complementary information from different imaging techniques; investigating the potential of federated learning to address data scarcity and privacy concerns; conducting prospective clinical trials to validate the impact of AI-guided HIFU on clinical outcomes, including symptom improvement, quality of life, and treatment cost-effectiveness; and establishing standardized reporting guidelines for AI applications in HIFU to facilitate meta-analyses and systematic reviews.

### 12.9. Summary of AI Use and HIFU Treatment for Uterine Fibroids

AI applications in HIFU treatment for uterine fibroids represent a rapidly evolving field with substantial potential to enhance treatment efficacy and safety. From patient selection to treatment monitoring and outcome prediction, AI offers valuable tools that complement clinical expertise. The various machine learning approaches, including gradient boosting, deep learning, and radiomics analysis, demonstrate complementary strengths that can be leveraged to address different challenges in HIFU treatment.

As these technologies continued to mature and undergo rigorous clinical validation, they were likely to become integral components of HIFU workflows, facilitating more personalized, efficient, and effective treatments for women with symptomatic uterine fibroids.

## 13. Embolization

Uterine fibroid embolization (UAE) is an established treatment option for symptomatic fibroids. Despite its clinical efficacy, predicting treatment outcomes remains challenging for clinicians. Luo et al. conducted a groundbreaking study [57] to develop and validate a deep learning model for predicting clinical outcomes before UAE procedures using routine MRI data.

### 13.1. Methodology and Model Architecture of the Study

Luo et al. [57] retrospectively analyzed 727 fibroids from 409 patients who underwent UAE at a single institution between 2007 and 2018. Their approach utilized a residual convolutional neural network (ResNet) architecture, which was particularly effective for processing hierarchical representations in imaging data. The model was trained on manually segmented fibroids from T1-weighted contrast-enhanced (T1C) and T2-weighted MRI sequences obtained before the procedure.

**Figure 6:**
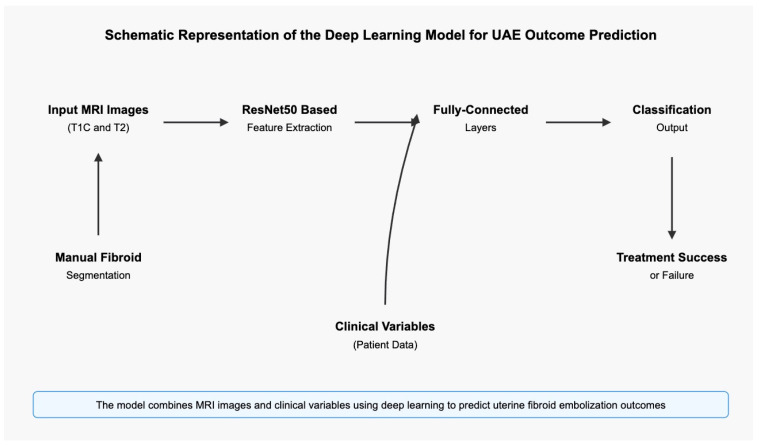
Schematic representation of the deep learning model for UAE outcome prediction.

The model architecture, shown in Figure 6, incorporated several sophisticated elements: a ResNet50 backbone with modified fully connected layers; image preprocessing with N4 bias correction; a 2.5-dimensional input approach using the largest axial, sagittal, and coronal slices; data augmentation techniques (horizontal flip, vertical flip, shear, and zoom transformations); and ensemble modeling that combined clinical variables with imaging features.

### 13.2. Performance and Clinical Significance of the Study

The study results [57] demonstrated the model’s robust predictive capabilities. At clinical follow-up, 85.6% of patients experienced symptom resolution or improvement, while 14.4% showed no improvement or worsening of symptoms.

As shown in Table 4, the performance of the T1C-trained model and the ensemble model surpassed that of human experts across most metrics, particularly in accuracy (0.847 vs. 0.722) and sensitivity (0.932 vs. 0.852). This highlights the potential of AI to enhance clinical decision-making in fibroid treatment planning.

### 13.3. Comparative Advantages and Study’ Limitations

Several of the study’s advantages [57] were identified: the model demonstrated significantly higher accuracy and sensitivity compared to experienced radiologists, potentially reducing subjective variability in treatment planning; the ensemble model incorporated both clinical variables and multiparametric MRI, providing a more comprehensive assessment; the researchers employed gradient-weighted class activation mapping to visualize the regions on which the algorithm focused, making the model more transparent and trustworthy for clinical implementation; and the model provided valuable prognostic information prior to an invasive procedure, potentially sparing patients from ineffective treatments.

However, limitations were also observed: the model was trained and tested on data from a single institution, raising concerns about generalizability across different patient populations and imaging protocols; while the model exhibited high sensitivity (0.932), its specificity (0.462) was considerably lower, suggesting a potential for overestimating treatment success; the reliance on manually segmented fibroids introduced potential operator variability and limited scalability for widespread clinical adoption; and, as with many AI applications in healthcare, issues related to data privacy, model interpretability, and algorithmic bias required careful consideration before clinical implementation.

### 13.4. Future Directions

The study by Luo et al. [57] represented a significant advancement in applying deep learning to predict UAE outcomes. Future research should focus on (a) multicenter validation with diverse patient populations; (b) the incorporation of automatic segmentation techniques and extension to other fibroid treatment modalities; and (c) the development of explainable AI approaches to increase clinical trust and adoption.

In conclusion, deep learning approaches show considerable promise for enhancing UAE treatment planning and patient selection. By accurately predicting which patients are likely to benefit from the procedure, these models could significantly improve clinical decision-making, reduce unnecessary procedures, and optimize healthcare resource utilization. However, further validation in diverse clinical settings and addressing current limitations remain necessary before widespread implementation.

## 14. Limitations and Challenges of AI in Fibroid Management

Despite the promising applications of artificial intelligence in the diagnosis and treatment of uterine fibroids, several significant limitations and challenges have been identified that must be addressed before widespread clinical implementation can be achieved. Some of these are reported in Table 5.

### 14.1. Model Interpretability

A major limitation of many AI models, particularly deep learning networks, is their “black box” nature. While these models achieve impressive diagnostic accuracy, the reasoning behind their decisions often remains opaque to clinicians. This lack of interpretability poses challenges in clinical settings, where understanding the rationale behind diagnostic and treatment recommendations is crucial for physician confidence and patient trust.

For instance, the DNN models described by Toyohara et al. [19] achieved high accuracy in differentiating between leiomyomas and sarcomas but offered limited insight into which imaging features most strongly influenced their classifications. Explainable AI approaches, such as attention maps and feature importance scores, were beginning to address this issue but remained underutilized in gynecological applications.

**Table 5:** AI models for uterine fibroid classification—performance comparison. PPV: Positive Predictive Value; NPV: Negative Predictive Value; AUC: area under the curve; AUROC: area under the receiver operating characteristic curve; HIFU: High-Intensity Focused Ultrasound; FPS: Frames Per Second; DCGAN: Deep Convolutional Generative Adversarial Network.

Study	Year	AI Method	Input Data Type	Sample Size	Performance Metrics	Advantages	Limitations
**Huo** **et al.** [29]	2023	Deep Convolutional Neural Network (DCNN)	Ultrasound images	3870 images (667 patients with UF, 570 without)	Accuracy: 94.72%, Sensitivity: 92.82%, Specificity: 97.05%, PPV: 97.45%, NPV: 91.73%	-Improved diagnostic capabilities of junior ultrasonographer -Performance comparable to senior ultrasonographer -Large sample size -Practical clinical application	-Single-center study -Limited demographic diversity -No external validation
**Yang** **et al.** [31]	2023	YOLOv3 (You Only Look Once, Version 3)	Ultrasound images	871 patients	Precision: 98.38%	-Real-time detection -High precision -Integration with existing ultrasound systems	-Limited evaluation metrics -No comparison with human experts -Single-center data -No reporting of false negatives
**Nakagawa et al.** [42]	2019	Machine Learning (six algorithms)	MRI (T2-weighted)	80 patients (30 uterine sarcoma, 50 benign leiomyoma)	AUC for eXtreme Gradient Boosting: 0.93	-Superior performance compared to radiologists -Multiple ML algorithms compared -Focus on T2-weighted MRI (widely available)	-Small sample size -Limited feature engineering -Single-center study
**Chiappa et al.** [28]	2021	Support Vector Machine and Bayesian analysis	Ultrasound with radiomics features	70 women (20 sarcoma, 50 fibroids)	Accuracy: 0.85, Sensitivity: 0.80, Specificity: 0.87, AUC: 0.86	-Combined radiomics features -Strong statistical approach -Good balance between sensitivity and specificity	-Single-center -Small sample size -No external validation -Potential selection bias
**Malek et al.** [44]	2019	Decision Tree Ensemble	Perfusion Weighted MRI	42 women (60 masses: 10 sarcomas, 50 benign)	Accuracy: 66.7%	-Novel use of perfusion imaging -Non-invasive approach -Multiple parameters analyzed	-Lower accuracy than other methods -Small sample size -Limited clinical validation
**Santoro et al.** [58]	2024	Machine Learning	CT-based radiomic features	Not explicitly stated in review	AUC: 0.78–0.82	-Use of CT (widely available) -Focused on differential diagnosis -Integration with clinical features	-Limited sample size -Single center -Lower sensitivity than MRI-based approaches
**Cai et al.** [59]	2024	Hybrid Deep Learning (MobileNetV2 + DCGAN)	Ultrasound images	871 patients	Accuracy: 97.45%, F1 score: 0.9741	-Real-time classification (40 FPS) -Data augmentation using GAN -Lightweight model for clinical deployment	-Single-center validation -Limited feature explanation -Single imaging modality
**Akpinar et al.** [50]	2022	Gradient Boosting Machine (GBM)	Multiparametric MRI	73 women	AUROC: 0.95, Accuracy: 0.92	-Incorporates multiple MRI features -High predictive performance -Focus on treatment outcome (HIFU)	-Relatively small sample -Complex feature engineering -Implementation complexity
**Luo et al.** [57]	2020	Residual Convolutional Neural Network (ResNet)	MRI (T1-weighted contrast)	Not explicitly stated	Accuracy: 0.847, Sensitivity: 0.932, Specificity: 0.462	-Prediction of embolization outcomes -Superior to radiologists -Easy to implement	-Imbalanced metrics (high sensitivity, low specificity) -Single center -Limited imaging protocols

### 14.2. Data Quality and Diversity

The performance of AI algorithms heavily depends on the quality and representativeness of the training data. Several limitations in current fibroid-related AI research include limited dataset size, data imbalance, demographic representation, and standardization issues. Many studies utilized relatively small datasets from single institutions, which may limit generalizability. For example, Chiappa et al. [28] developed their radiomics model using data from only one center, introducing potential selection bias.

The rarity of certain conditions, particularly uterine sarcomas compared to benign fibroids, creates inherent imbalances in training datasets that could bias AI algorithms toward the majority class. Most studies fail to include diverse patient populations across different ethnicities, ages, and comorbidity profiles, limiting the generalizability of AI models to the broader population of women with fibroids.

Variations in imaging acquisition parameters, scanner types, and protocols across different institutions complicate the development of robust AI models that perform consistently across diverse clinical settings.

### 14.3. Technical and Clinical Validation

A significant challenge in the field is the lack of rigorous validation protocols: many studies relied solely on internal validation methods (e.g., cross-validation) without testing their models on truly independent external datasets from different institutions; most current studies are retrospective in nature, with few prospective trials validating AI performance in real-time clinical settings; and the gold standard for diagnosis (histopathology) is often unavailable for all cases, particularly in non-surgical management scenarios, potentially introducing verification bias.

### 14.4. Performance on Rare and Edge Cases

**Figure 7:**
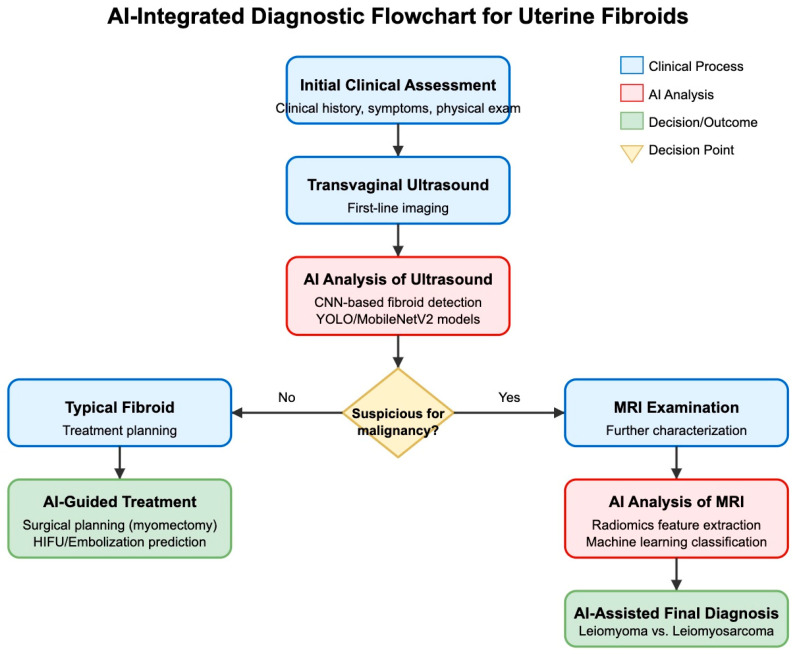
AI-integrated diagnostic flowchart for uterine fibroids.

AI models often struggle with rare presentations or atypical manifestations of fibroids. For this reason, an AI-integrated diagnostic flowchart for uterine fibroids could be proposed (Figure 7). Models trained primarily on typical fibroid cases might underperform when encountering unusual variants or degeneration patterns. The low prevalence of uterine sarcomas makes it difficult to develop algorithms with high sensitivity for these critical cases, as noted in the comparative study by Santoro et al. [58]. Additionally, the presence of comorbid gynecological conditions (e.g., adenomyosis, endometriosis) might confound AI algorithm performance in ways that remain poorly characterized.

### 14.5. Integration into the Clinical Workflow

Practical challenges to clinical integration are as follows: the implementation of AI systems requires substantial computational resources and technical expertise that might not be available in all clinical settings; integration with existing hospital information systems and PACS remains challenging; and clinicians require training to appropriately interpret and utilize AI-generated recommendations, adding an additional burden to already busy clinical practices.

### 14.6. Ethical and Regulatory Considerations

Important ethical and regulatory challenges are as follows: The use of large datasets for AI training raises important questions about patient privacy and consent, particularly when retrospective data is used. The rapidly evolving nature of AI algorithms poses challenges for traditional regulatory frameworks, leading to questions about when and how algorithms should be re-evaluated after deployment. Uncertainty remains concerning responsibility for errors in AI-assisted diagnoses and treatment planning. Finally, ensuring that AI advances benefit diverse patient populations equitably, rather than exacerbating existing healthcare disparities, remains a critical ethical consideration.

### 14.7. Cost-Effectiveness

The economic impact of AI implementation in fibroid management has remained largely unevaluated for the following reasons: (1) the substantial resources required for AI development and validation must be balanced against potential clinical benefits; (2) the infrastructure, training, and maintenance expenses associated with AI systems might be prohibitive for some healthcare settings; and (3) the long-term cost-effectiveness of AI applications in fibroid management, including potential reductions in unnecessary procedures or improved outcomes, requires further study.

Addressing these limitations requires collaborative efforts among radiologists, gynecologists, AI researchers, ethicists, and regulatory bodies to develop more robust, interpretable, and clinically validated AI solutions for fibroid management.

## 15. Conclusions

Creating classification models that differentiate between normal and pathological instances, as well as constructing models that automatically segment or measure ovarian volume or follicles, were the primary goals of the published literature on AI applied to ultrasound in benign gynecological illnesses [60].

To sum up, the precise identification of sarcomas and uterine fibroids is still a difficult task in clinical practice. Despite its high sensitivity, transvaginal ultrasonography may not detect all forms of fibroids [59]. Therefore, for a thorough assessment, additional imaging modalities such as MRI and hysteroscopy may be required [61,62]. Because malignant leiomyosarcomas and benign leiomyomas differ in their prognosis and methods of therapy, it is very important to distinguish between the two, including with AI support [58]. AI has started to show promise as a useful tool for enhancing diagnostic precision and directing therapeutic choices, especially in cases of rapidly growing fibroids [63].

When applied to ultrasound and MRI images, radiomics, machine learning, and DNN models show potential in distinguishing benign from malignant uterine lesions, thereby supporting gynecologic oncologists in their clinical decision-making [64]. These AI-powered methods provide insightful decision support, especially in situations in which the diagnosis is unclear and in cases in which only ultrasound is available due to the lack of more complex instruments, such as MRI [65].

AI has transformed surgical operations, including robotic-assisted laparoscopic surgery, HIFU ablation, and myomectomy. AI-powered solutions have increased surgical accuracy, made it easier to identify structures in real time, and improved patient outcomes [66]. Surgeons could limit the risk of complications and optimize treatment procedures by utilizing deep learning algorithms and modern imaging techniques. Additionally, AI was critical in tracking progress during operations such as uterine fibroid embolization and predicting treatment outcomes. Promising accuracy in predicting clinical outcomes has been demonstrated by deep learning models trained on pre-procedure MRI scans, providing doctors with useful insights into patient responses to therapy.

Overall, gynecology and gyneco-oncology have advanced significantly with the use of artificial intelligence in the diagnosis and treatment of uterine fibroids and sarcomas. AI technologies have the ability to significantly improve patient care, increase the accuracy of diagnoses, and simplify treatment plans in this intricate clinical environment as they develop and receive additional validation. However, implementation challenges must be addressed through collaborative research efforts before widespread clinical adoption.

Future research should focus on developing multi-institutional datasets (with diverse patient populations) and on creating more interpretable AI models that provide insight into their decision processes. Researchers should conduct prospective clinical trials to validate AI performance in real-world settings, addressing ethical and regulatory considerations for responsible AI implementation. Finally, the cost-effectiveness of AI integration into fibroid management should be fully evaluated, including an exploration of hybrid approaches that combine the strengths of different AI methodologies.

The integration of AI into fibroid diagnosis and management holds great promise but requires continued rigorous evaluation and refinement to ensure that these technologies truly enhance patient care while minimizing potential risks and limitations.

## Figures and Tables

**Table 1:** Comparison of key AI studies on uterine fibroid diagnosis.

Study	Approach	Sample Size	Primary Objective	Key Performance	Main Limitations
**Chiappa et al. (2021)** [28]	Radiomics with machine learning	70 patients (20 sarcomas, 50 fibroids)	Differentiate sarcomas from fibroids	Accuracy: 0.85, Sensitivity: 0.80, Specificity: 0.87, AUC: 0.86	Single-center, small sample size, limited external validation
**Huo et al. (2023)** [29]	Deep Convolutional Neural Network	3870 images from 1237 patients	Enhance junior ultrasonographer performance	Accuracy: 94.72%, Sensitivity: 92.82%, Specificity: 97.05%	“Black box” model, limited interpretability, focuses only on fibroid detection
**Yang et al. (2023)** [31]	YOLOv3 adaptation	Not specified in review	Real-time fibroid detection	Precision: 98.38%	Limited information on validation methodology, unknown generalizability

**Table 2:** Comparison of AI-powered diagnostic methods for uterine fibroids. US: ultrasound; MRI: magnetic resonance imaging; PWI: perfusion weighted imaging; DCNN: deep convolutional neural network; DNN: deep neural network.

Method	Study	Diagnostic Approach	Sample Size	Key Technology	Performance Metrics	Clinical Application	Key Advantages	Limitations
**Radiomics with US**	Chiappa et al. [28]	Feature extraction from ultrasound images	70 patients (20 sarcomas, 50 fibroids)	Support Vector Machines, Random Forests, kNNs	Accuracy: 0.85 ± 0.01. Sensitivity: 0.80 ± 0.01. Specificity: 0.87 ± 0.01. AUC: 0.86 ± 0.03	Preoperative differentiation of sarcomas from fibroids	Interpretable features; Uses widely available ultrasound	Small sample size; Single-center data; Limited validation
**DCNN with US**	Huo et al. [29]	Deep learning on ultrasound images	3870 ultrasound images from 1237 patients	Deep Convolutional Neural Network	Accuracy: 94.72%; Sensitivity: 92.82%; Specificity: 97.05%	Assisting junior ultrasonographer in fibroid detection	Elevates junior practitioner performance to match specialists; Large dataset	“Black box” model; Focuses only on fibroid detection, not sarcoma differentiation
**DNN with MRI**	Toyohara et al. [19]	Deep learning on multiple MRI sequences	263 patients (63 sarcomas, 200 fibroids)	MobileNet-V2 neural network	Accuracy: 90.3%; Sensitivity: 89.8%; Specificity: 91.7%	Preoperative diagnosis of uterine sarcomas	Comparable to radiological specialists; Multiple MRI sequence integration	Limited transferability to different imaging protocols; High computational requirements
**PWI with MRI**	Malek et al. [44]	Machine learning on perfusion parameters	42 women with 60 masses (10 sarcomas, 50 fibroids)	Decision Tree Ensemble	Sensitivity: 100%; Specificity: 90% at optimal operating point	Differentiation of sarcomas from fibroids using PWI parameters	Novel approach using perfusion characteristics; High sensitivity	Very small sample size; Complex acquisition protocol
**Haptic Interface**	Doria et al. [45]	Cutaneous softness rendering for palpation	13 gynecologic surgeons evaluating 4 specimen types	Wearable Fabric Yielding Display (W-FYD)	Accuracy: 74.8% (vs. 83.8% with direct palpation); No statistically significant difference	Intraoperative fibroid localization during robot-assisted surgery	Integration with surgical workflow; Restores tactile feedback; Real-time assessment	Integration complexity with existing surgical robots; Requires specialized hardware; Needs sensor miniaturization

**Table 3:** Performance of machine learning algorithms in HIFU outcome prediction.

Algorithm	AUROC	Accuracy	Specificity	Sensitivity
**Gradient Boosting (GBM)**	0.95	0.92	1.00	0.89
**Random Forest**	0.92	0.96	0.83	1.00
**AdaBoost**	0.92	0.88	0.83	0.89
**Logistic Regression**	0.83	0.84	0.66	0.89
**Support Vector Machine**	0.78	0.84	0.50	0.94

**Table 4:** Performance comparison of AI models vs. radiologists in predicting UAE outcomes. Values in parentheses represent the 95% confidence interval range. PPV = Positive Predictive Value; NPV = Negative Predictive Value; T1C = T1-weighted contrast-enhanced.

Performance Metric	T1C Model	T2 Model	Ensemble Model	Average of 4 Radiologists
**Accuracy (95% CI)**	0.847 (0.745–0.914)	0.806 (0.698–0.882)	0.847 (0.745–0.914)	0.722 (0.609–0.813)
**Sensitivity (95% CI)**	0.932 (0.833–0.978)	0.932 (0.833–0.978)	0.966 (0.878–0.997)	0.852 (0.737–0.923)
**Specificity (95% CI)**	0.462 (0.232–0.709)	0.231 (0.075–0.509)	0.308 (0.124–0.580)	0.135 (0.021–0.415)
**PPV**	0.887	0.846	0.864	0.831
**NPV**	0.600	0.429	0.667	0.231

## Abbreviations

ML	Machine learning
AI	Artificial intelligence
US	Ultrasound
MMT	Malignant mesenchymal tumors
WHO	World Health Organization
LMS	Leiomyosarcoma
LG-ESS	Low-grade endometrial stromal sarcoma
HG-ESS	High-grade endometrial stromal sarcoma
UUS	Undifferentiated uterine sarcoma
AS	Adenosarcoma
STUMP	Smooth muscle tumors of unknown malignant potential
LDH	Lactate dehydrogenase
MRI	Magnetic resonance imaging
mp-MRI	Multiparametric MRI
DCNN	Deep convolutional neural network
YOLOv3	You Only Look Once, Version 3
PACS	Picture Archiving and Communication System
SWI	Susceptibility-weighted imaging
DWI	High diffusion-weighted imaging
ADC	Low apparent diffusion coefficient
AUCs	Areas under the curve
DNN	Deep neural network
ROIs	Regions of interest
LOOCV	Leave-one-out cross-validation
RMIS	Robot-Assisted Minimally Invasive Surgery
FCNN	Convolutional neural network
HIFU	High-Intensity Focused Ultrasound
NPV	Non-Perfused Volume
GBM	Gradient Boosting Machine
CE-T1WI	Contrast-enhanced t1-weighted imaging
LASSO	Least Absolute Shrinkage and Selection Operator
T2WI	Combined T2-Weighted Imaging
SVM	Support Vector Machine
RF	Random forest
LightGBM	Light Gradient Boosting Machine
MLP	Multi-Layer Perceptron
SR-DWI	Super-Resolution Diffusion-Weighted Imaging
ResNet	Residual convolutional neural network
